# The mutational burden and oligogenic inheritance in Klippel-Feil syndrome

**DOI:** 10.1186/s12891-020-03229-x

**Published:** 2020-04-11

**Authors:** Ziquan Li, Sen Zhao, Siyi Cai, Yuanqiang Zhang, Lianlei Wang, Yuchen Niu, Xiaoxin Li, Jianhua Hu, Jingdan Chen, Shengru Wang, Huizi Wang, Gang Liu, Ye Tian, Zhihong Wu, Terry Jianguo Zhang, DISCO (Deciphering Disorders Involving Scoliosis and COmorbidities) study, Yipeng Wang, Nan Wu

**Affiliations:** 1 Department of Orthopedic Surgery, Peking Union Medical College Hospital, Peking Union Medical College and Chinese Academy of Medical Sciences, Beijing, 100730 China; 2 Beijing Key Laboratory for Genetic Research of Skeletal Deformity, Beijing, 100730 China; 3 Graduate School of Peking Union Medical College, Beijing, 100005 China; 4 Medical Research Center, Peking Union Medical College Hospital, Peking Union Medical College and Chinese Academy of Medical Sciences, Beijing, 100730 China; 5 Key Laboratory of Big Data for Spinal Deformities, Chinese Academy of Medical Sciences, Beijing, 100730 China

**Keywords:** Klippel-Feil syndrome, Whole-exome sequencing, Genetic burden analysis, Genetic mutational spectrum, Oligogenic inheritance

## Abstract

**Background:**

Klippel-Feil syndrome (KFS) represents a rare anomaly characterized by congenital fusion of the cervical vertebrae. The underlying molecular etiology remains largely unknown because of the genetic and phenotypic heterogeneity.

**Methods:**

We consecutively recruited a Chinese cohort of 37 patients with KFS. The clinical manifestations and radiological assessments were analyzed and whole-exome sequencing (WES) was performed. Additionally, rare variants in KFS cases and controls were compared using genetic burden analysis.

**Results:**

We primarily examined rare variants in five reported genes (*GDF6*, *MEOX1, GDF3, MYO18B* and *RIPPLY2*) associated with KFS and detected three variants of uncertain significance in *MYO18B*. Based on rare variant burden analysis of 96 candidate genes related to vertebral segmentation defects, we identified *BAZ1B* as having the highest probability of association with KFS, followed by *FREM2*, *SUFU*, *VANGL1* and *KMT2D*. In addition, seven patients were proposed to show potential oligogenic inheritance involving more than one variants in candidate genes, the frequency of which was significantly higher than that in the in-house controls.

**Conclusions:**

Our study presents an exome-sequenced cohort and identifies five novel genes potentially associated with KFS, extending the spectrum of known mutations contributing to this syndrome. Furthermore, the genetic burden analysis provides further evidence for potential oligogenic inheritance of KFS.

## Background

Klippel-Feil syndrome (KFS), which was first reported by Maurice Klippel and Andre Feil in 1912, is a complex skeletal disorder characterized by congenital fusion of two or more cervical vertebrae [1]. The incidence of KFS has been estimated as approximately 1/40000–1/42000, with a slight female predominance by approximately 3:2 [2, 3]. However, the true incidence of KFS may be higher than reported because of heterogeneity in phenotypic expression and a lack of large-scale population screening studies. Based on retrospective studies of cervical computed tomography (CT) in 2917 patients and 131 patients with cervical spondylotic myelopathy, the incidence of KFS may be as high as 1/172 to 5/131 [4, 5].

The main etiology of KFS is the impaired development of the cervical vertebrae, which leads to the improper segmentation of the cervical spine. Although KFS can occur as an isolated malformation, it is often associated with a variety of congenital diseases and other systemic malformations, including scoliosis, Sprengel deformity, urinary malformations, gastrointestinal malformations, hearing impairment, congenital heart defects and various neurological anomalies [1, 6–8]. In addition, congenital fusion deformity of the cervical vertebrae often alters the kinematics of the cervical spine in ways that may accelerate degenerative changes throughout the region. Therefore, patients with KFS usually complain of neck and back pain, respiratory failure, and decreased mobility and may be at risk for death in severe cases due to a lack of early prediction methods.

Recent studies have suggested that the failed vertebral segmentation underlying KFS is caused by defective somitogenesis in the cervical region [9]. Autosomal dominant and recessive inheritance patterns have both been reported in families with KFS. According to Online Mendelian Inheritance in Man (OMIM), mutations in *GDF6* (MIM: 601147), *MEOX1* (MIM: 600147), *GDF3* (MIM: 606522), *MYO18B* (MIM: 607295) and *RIPPLY2* (MIM: 609891) have been associated with KFS [10–15]. Nevertheless, the basis of genetic predisposition to KFS is largely unknown due to the rarity of the disease; only a small number of KFS cases can be explained by the five specific pathogenic genes mentioned above [16]. To further decipher the molecular basis of KFS at the exome level, we herein investigate the molecular findings of WES among a cohort of 37 KFS patients/families, and further analyze rare variants by using a genetic burden method.

## Methods

### Cohort collection

From January 2016 to April 2018, we consecutively recruited 37 patients of Han Chinese ethnicity at Peking Union Medical College Hospital (PUMCH) who had been diagnosed with KFS under DISCO (Deciphering disorders Involving Scoliosis and COmorbidities, DISCO http://discostudy.org/) project. Demographic information, physical examination results, clinical symptoms on presentation, and a detailed medical history were obtained. Radiological assessments including anteroposterior, lateral neutral, and flexion-extension plain radiographs; CT; and magnetic resonance imaging (MRI) were performed on each patient to give a prior clinical diagnosis. Radiographic parameters of interest, such as the segmentation of congenitally fused vertebrae, the Samartzis classification of KFS [8], cervical scoliosis, and sagittal cervical alignment, were also recorded. All radiographic evaluations were conducted by trained spine surgeons, and the clinical review was performed by an alternate observer blinded to the radiographic assessment. None of the investigators were involved in the direct care of the patients.

Prior to study participation, written informed consent was provided by each participant. The study was approved by the Department of Scientific Research and Ethics Committee of PUMCH in China.

### Genomic DNA preparation and whole-exome sequencing

Genomic DNA for 37 KFS patients (14 cases along with their healthy parents) was extracted from peripheral blood lymphocytes. WES was performed on peripheral blood DNA for all participants. DNA samples were prepared in Illumina libraries and then underwent whole-exome capture with the SureSelect Human All Exon V6 + UTR r2 core design (91 Mb, Agilent, USA), followed by sequencing on the Illumina HiSeq 4000 platform in 150-bp paired-end reads mode (Illumina, San Diego, CA, USA).

### Variant annotation and interpretation

WES data processing was performed with the in-house developed PUMP (Peking Union Medical college hospital Pipeline) [17]. Interpretation of single-nucleotide variants (SNVs) and insertions/deletions (indel variant alleles) were adapted from the American College of Medical Genetics and Genomics (ACMG) guidelines [18]. Computational prediction tools (Genomic Evolutionary Rate Profiling [GERP] [19], Combined Annotation Dependent Depletion [CADD] [20], Sorting Intolerant Form Tolerant [SIFT] [21], Polyphen-2 [22], and MutationTaster [23]) were used to predict the conservation and pathogenicity of candidate variants. All variants were compared to publicly available databases such as the 1000 Genomes Project (http://www.internationalgenome.org/), the NHLBI GO Exome Sequencing Project (ESP) Exome Variant Server (http://evs.gs.washington.edu/EVS/), and the Exome Aggregation Consortium (ExAC) database (http://exac.broadinstitute.org/). The in-house control database consisted of WES data from 534 unrelated Chinese individuals with no apparent skeletal anomalies from DISCO project.

### Mutational burden analysis

A criterion of the internal (combined case and control population) and external (ExAC, and gnomAD) was used to filter for rare genuine variants with a minor allele frequency (MAF) < 0.001.

After filtering, we performed an initial analysis focusing on variants in reported pathogenic genes (*GDF6*, *MEOX1, GDF3, MYO18B* and *RIPPLY2*) related to KFS. Additionally, 96 genes that were biologically related to vertebral segmentation defects according to previous reports or the Human Phenotype Ontology (HPO; https://hpo.jax.org/), NCBI Gene (https://ncbi.nlm.nih.gov/gene/) and OMIM (http://omim.org/) databases were added to the candidate gene list (Table S1).

To extend the spectrum of known mutations contributing to KFS, we performed a second-stage analysis to search for exome-wide rare variants in 96 candidate genes related to vertebral segmentation defects using the genetic burden analysis [24].

### Statistical analysis

Mean comparison of relevant features was conducted using Student’s t-test. One-tailed *P*-values of < 0.05 were considered statistically significant. Fisher’s exact test was used for genetic burden analysis.

## Results

### Cohort information

The study cohort consisted of 37 unrelated KFS patients of Han Chinese ethnicity, including 14 cases with unaffected parents (trios) and 23 singleton cases (Table S2). There were 20 males (54.1%) and 17 females (45.9%), with a mean age at diagnosis of 12.6 ± 5.3 years. Detailed demographic information is presented in Table 1.

**Table 1:** Demographic and clinical characteristics of the KFS cohort

Parameter	KFS cohort (*n* = 37)
**Age in years, mean (range)**	12.6 (5–26)
**Sex, n (%)**	
Men	20 (54.1)
Women	17 (45.9)
**Level of fusion, n (%)**	
C1–C2	3 (8.1)
C2–C3	15 (40.5)
C3–C4	14 (37.8)
C4–C5	10 (27.0)
C5–C6	13 (35.1)
C6–C7	21 (56.8)
C7–T1	10 (27.0)
**KFS classification, n (%)**	
Type I	15 (40.5)
Type II	5 (13.5)
Type III	17 (45.9)
**Comorbidities, n (%)**	
Intraspinal anomalies	9 (24.3)
Extraskeletal anomalies	12 (32.4)
**Clinical manifestations, n (%)**	
Limited cervical ROM	17 (45.9)
Short neck	10 (27.0)
Low posterior hairline	6 (16.2)
Clinical triad	4 (10.8)
Torticollis	4 (10.8)

Abbreviation: *ROM* range of motion

### Clinical features and radiographic parameters

**Fig. 1:**
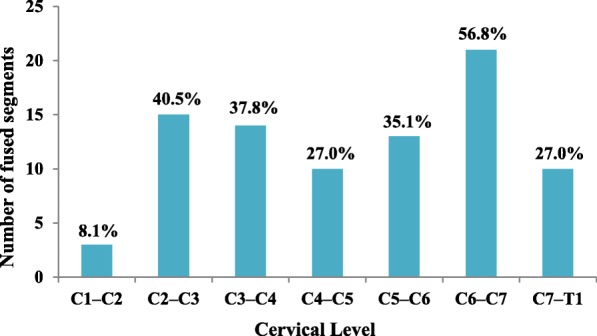
The number and prevalence (%) of fused segments in our KFS cohort. The figure illustrates the distribution of fused cervical levels among the cervical segments

Among the 37 patients with KFS, there was a mean of 2.3 congenital fusion levels (range 1–7), which included various regions of the cervical spine. The most commonly fused segments were C6–C7, C2–C3, and C3–C4, which occurred in 56.8%, 40.5%, and 37.8% of the patients, respectively (Fig. 1). According to the KFS classification criteria by Samartzis [8], 15 (40.5%) patients were Type I, with a single congenitally fused cervical segment; five (13.5%) patients were Type II, demonstrating multiple noncontiguous congenitally fused cervical segments; and 17 (45.9%) patients were Type III, showing multiple contiguous congenitally fused cervical segments.

The KFS patients presented with varied intraspinal and extraskeletal anomalies. There were nine patients (24.3%) manifesting intraspinal deformities, including syringomyelia, diastematomyelia, and tethered cord, and 12 patients (32.4%) presented with extraskeletal manifestations such as various cardiac defects and malformation of the urinary and digestive systems. KFS has been characterized as a clinical triad of short neck, low posterior hairline, and limited cervical range of motion (ROM). In our cohort, limited cervical ROM was the most common clinical feature (17 patients, 45.9%), followed by short neck (10 patients, 27.0%) and low posterior hairline (six patients, 16.2%). However, 43.2% of the patients did not show any typical manifestation of the clinical triad; furthermore, only 10.8% of the patients showed all three signs in the clinical triad at the same time. Detailed characteristics and radiographic parameters are shown in Table 1.

### Variants of known KFS-related genes

After WES data processing and variant interpretation, we firstly examined filtered rare variants for potential causative variants in five reported genes associated with KFS. As a result, 3 variants were found in *MYO18B* (Table 2): a splice region mutation (c.2695 + 3A > G) and two missense variants (c.662 T > C and c.5020G > A) were identified in patients CS132, CS1015 and CS1049, respectively. The c.2695 + 3A > G variant was found in patient CS132, who was affected by cervical vertebral fusion (C2–C6), short neck, low posterior hairline, limited cervical ROM and protruding ears but no rib or spinal cord abnormalities. Moreover, congenital cervical fusions combined with congenital heart disease were found in both CS1015 and CS1049. The variant c.662 T > C (p. Leu221Pro) in exon 4 was identified in proband CS1015, who presented with a single vertebral fusion level at C6–C7 and no severe clinical manifestations in the cervical spine. However, the c.5020G > A (p. Gly1674Arg) variant was observed in exon 31 from patient CS1049, who had long-segment cervical fusion (C1-T1), a prominent clinical triad and torticollis.

**Table 2:** *MYO18B* variants and clinical features of patients

Identifier	CS132	CS1015	CS1049
Sex/age (years) at diagnosis	F/12	M/5	F/7
Mutation information			
Variant type	Splice region	Missense	Missense
Zygosity	Heterozygous	Heterozygous	Heterozygous
Chr_Position	22_26219648	22_26164545	22_26299670
Variant nomenclature	c.2695 + 3A > G	c.662 T > C (p.Leu221Pro)	c.5020G > A (p.Gly1674Arg)
ExAC AF	0.000017	0.000034	0.000013
gnomAD AF	Novel	0.0000324	0.00003232
In-house exome database AF	0.0029	Novel	Novel
Clinical features			
Fused levels	C2–C6	C6–C7	C1-T1
Comorbidities	Protruding ears	Tetralogy of Fallot, congenital solitary kidney	Patent foramen ovale
Clinical manifestations	Clinical triad	None	Clinical triad, torticollis
Classification	Type III	Type I	Type III
Other vertebral and costal abnormalities	None	None	Rib fusion Sacrococcygeal agenesis

MYO18B, an unconventional class XVIII myosin, is mainly expressed in human cardiac and skeletal muscle and plays putative roles in diverse human syndromes and cellular processes [25]. Consistent with the reported phenotypes of myocardial defects in *MYO18B*-deficient mice, two of our patients suffered from cardiac deformities. A homozygous nonsense mutation in *MYO18B* (c.6905C > A: p.S2302*) was identified in two unrelated patients who came from consanguineous families and exhibited a similar phenotype of KFS, myopathy, short stature, and facial dysmorphism [14], and moreover, compound heterozygous frameshift variants (c.6768delG: p.Leu2257SerfsTer16 and c.6660_6670delATTAGAACCTG: p.Arg2220SerfsTer74) in *MYO18B* were reported in a medulloblastoma patient with a previous diagnosis of KFS [26]; nonetheless, there are still no reports on pathogenic missense variants in *MYO18B* associated with KFS due to the limited sample size.

### Genetic burden analysis of candidate genes related to vertebral segmentation defects

Based on the rare variant filtering of the candidate gene list (Table S1), we found that the average number of rare variants per individual in the KFS cohort (*n* = 33) was significantly higher than that in control individuals (*n* = 202) (*p* < 0.0001) (Table S3), which suggests that the candidate genes were relevant to the KFS etiology.

Furthermore, in the gene-based burden analysis, we compared the exome-wide frequency of rare coding variants for each candidate gene in KFS cases and controls. Among the genes associated with the KFS cohort compared to controls, the five with the lowest *P*-values were *BAZ1B* (*P* = 0.00000002), *FREM2* (*P* = 0.0003), *SUFU* (*P* = 0.0004), *VANGL1* (*P* = 0.0072) and *KMT2D* (*P* = 0.0214) (Table 3).

**Table 3:** Genetic burden analysis for rare variant frequencies in KFS cases and controls

	KFS cases (n)	Variant alleles	Control subjects (n)	Variant alleles	*p*-value
*BAZ1B*	37	3	534	1	0.00000002
*FREM2*	37	3	534	5	0.0003
*SUFU*	37	2	534	2	0.0004
*VANGL1*	37	2	534	4	0.0072
*KMT2D*	37	3	534	11	0.0214

### Variants identified in potential KFS-associated genes

We identified three qualified variants of *BAZ1B* from three patients. The in-frame insertion variant c.3804_3821dupGGAGGAGGAGGAAGAAGA (p. Glu1268_Glu1273dup) in *BAZ1B* was identified in CS63. The patient was a 6-year-old female with complaints of restricted neck motion and torticollis. Radiological evaluation detected fusion of the cervical vertebrae at C6-T1, scoliosis, diastematomyelia, syringomyelia, and polycystic kidney disease. *BAZ1B* encodes a member of a bromodomain protein family that is involved in chromatin-dependent regulation of transcription. Deletion of this gene has been reported in Williams-Beuren syndrome (WBS), a developmental disorder featuring multiple skeletal deformities such as scoliosis, hallux valgus, little-finger clinodactyly, fusion of the cervical spine and Chiari I malformation [27]. Although *BAZ1B* has no previously identified connection to KFS, the present finding may represent a novel disease association. In addition, two other rare missense variants of *BAZ1B*, namely, c.527A > G (p.Lys176Arg) and c.1364G > A (p.Arg455Gln), were also identified. Both patients suffered from single-segment cervical fusion and only mild clinical manifestations, despite multiple intraspinal deformities such as diastematomyelia, tethered cord and syringomyelia.

A heterozygous missense variant of c.8479C > T (p. Arg2827Cys) in the *FREM2* gene was detected, with an ExAC allele frequency of 0.000016. This variant was predicted as pathogenic by the functional prediction programs SIFT, Polyphen-2 and MutationTaster, with a GERP score of 3.79 and a CADD score of 18.07. It was reported that homozygosity for a splice site mutation in the *FREM2* gene was associated with Fraser syndrome and that compound heterozygosity for a missense mutation was related to cryptophthalmos [28, 29].

Three other rare heterozygous missense variants c.13364G > A (p.Arg4455His), c.3074C > T (p.Ser1025Leu), c.8939C > T (p.Ala2980Val) in *KMT2D* were identified in CS488, CS1162 and CS1210. These three patients suffered from multiple contiguous fused cervical levels: C2–C7, C2–C7, and C5-T1, respectively. The protein encoded by *KMT2D* has been shown to be a transcriptional regulator of the beta-globin and estrogen receptor genes and is reported to be a cause of Kabuki syndrome [30–32]. Therefore, the significant association of *KMT2D* with KFS involving multiple fused segments (Type III) may represent an expansion of the known phenotype associated with this gene.

Two other rare missense variants in *FREM2* and variants of the associated genes *VANGL1* and *SUFU* are shown in Table 4.

**Table 4:** Information on rare variants of five novel associated genes

Patient	Gene symbol	Variant type	Zygosity	Chr_Position	Ref transcript	Variant nomenclature	GERP++ score	CADD score	ExAC AF-total
CS63	*BAZ1B*	In-frame insertion	Het	7_72861616	NM_032408.3	c.3804_3821dupGGAGGAGGAGGAAGAAGA (p.Glu1268_Glu1273dup)	–	–	0.000016
CS216	*BAZ1B*	Missense	Het	7_72912871	NM_032408.3	c.527A > G (p.Lys176Arg)	5.26	17.03	0.0000082
CS519	*BAZ1B*	Missense	Het	7_72892427	NM_032408.3	c.1364G > A (p.Arg455Gln)	5.56	11.17	0.000016
CS193	*FREM2*	Missense	Het	13_39452441	NM_207361.4	c.8842 T > C (p.Tyr2948His)	0.572	5.468	0.000033
CS1049	*FREM2*	Missense	Het	13_39450454	NM_207361.4	c.8479C > T (p.Arg2827Cys)	3.79	18.07	0.000016
OS1056	*FREM2*	Missense	Het	13_39424205	NM_207361.4	c.6410A > T (p.Tyr2137Phe)	5.79	17.11	0.0000083
CS488	*KMT2D*	Missense	Het	12_49425124	NM_003482.3	c.13364G > A (p.Arg4455His)	5.57	13.45	0.000058
CS1162	*KMT2D*	Missense	Het	12_49444297	NM_003482.3	c.3074C > T (p.Ser1025Leu)	3.4	6.896	0.000059
CS1210	*KMT2D*	Missense	Het	12_49432200	NM_003482.3	c.8939C > T (p.Ala2980Val)	2.77	11.24	0.000017
CS63	*SUFU*	Missense	Het	10_104375107	NM_016169.3	c.1105G > A (p.Val369Ile)	5.21	12.54	0.000091
CS132	*SUFU*	Splice region	Het	10_104375165	NM_016169.3	c.1157 + 6C > T	–	–	0.000033
CS1130	*VANGL1*	Synonymous	Het	1_116206326	NM_138959.2	c.249G > A (p.Ser83=)	–	–	0.00012
CS927	*VANGL1*	Missense	Het	1_116227985	NM_138959.2	c.1151C > G (p.Pro384Arg)	5.44	25.8	0.0000083

Abbreviations: *Het* heterozygous, *Chr* chromosome, *ExAC* Exome Aggregation Consortium

### Potential oligogenic inheritance

Among qualified variants, seven patients (18.9%) showed potential involvement of multiple associated variations in different genes (Table 5), which represented an increased ratio compared with that of the in-house controls (30/534) (*P* = 0.00148). Specifically, an in-frame insertion variant of *BAZ1B*, a splice acceptor variant of *GRIP1*, and two missense variants of *SUFU* and *TBX6* were detected in patient CS63, and there were three patients carrying three variants in different candidate genes and another three patients carrying two variants in different genes. Additionally, *MYO18B, SUFU,* and *BAZ1B*, mutations of which were probably causative of KFS, were identified in two or more patients. This finding suggests the possibility of an oligogenic pathogenesis pattern.

**Table 5:** Potential oligogenic inheritance in seven KFS patients

Patient	Gene symbol	Variant type	Variant nomenclature
CS63	*BAZ1B*	In-frame insertion	c.3804_3821dupGGAGGAGGAGGAAGAAGA (p.Glu1268_Glu1273dup)
	*GRIP1*	Splice acceptor	c.1043-1G > A
	*SUFU*	Missense	c.1105G > A (p.Val369Ile)
	*TBX6*	Missense	c.499C > T (p.Arg167Cys)
CS132	*MYO18B*	Splice region	c.2695 + 3A > G
	*SUFU*	Splice region	c.1157 + 6C > T
	*WNT7A*	Missense	c.83C > T (p.Ser28Leu)
CS587	*FUZ*	Missense	c.819C > A (p.Asp273Glu)
	*MAP3K7*	Missense	c.1115G > A (p.Arg372His)
	*POR*	Missense	c.1798C > T (p.Arg600Trp)
CS676	*CHD7*	Synonymous	c.4008C > T (p.Ile1336=)
	*FRAS1*	Missense	c.7423G > A (p.Glu2475Lys)
CS519	*BAZ1B*	Missense	c.1364G > A (p.Arg455Gln)
	*COG1*	Missense	c.739C > T (p.His247Tyr)
CS1015	*ANKRD11*	Missense	c.6067G > T (p.Ala2023Ser)
	*HOXD13*	Missense	c.814G > A (p.Val272Ile)
	*MYO18B*	Missense	c.662 T > C (p.Leu221Pro)
CS1049	*FREM2*	Missense	c.8479C > T (p.Arg2827Cys)
	*MYO18B*	Missense	c.5020G > A (p.Gly1674Arg)

## Discussion

KFS is a relatively rare disorder characterized by congenital synostosis of the cervical vertebrae because of a segmentation or formation defect. However, articles related to KFS are limited and mostly use individual case reports or small case series. Therefore, the 37-patient cohort in the current study is a relatively large reported KFS cohort for assessing clinical features and radiologic parameters and for identifying molecular findings by WES.

The clinical manifestations of KFS show substantial heterogeneity, and the typical clinical features are defined as a triad of short neck, low posterior hairline, and limited neck ROM. However, as found in our case cohort, the complete clinical triad was noted in only 10.8% of patients. Moreover, 43.2% of our KFS cohort did not present with any of these findings, which was consistent with a single-center retrospective study of 31 patients by Samartzis in 2016 [33]. Further analysis with imaging data demonstrated that 73.3% of the 15 Type I KFS patients exhibited none of the three clinical triad signs; on the other hand, all four patients presenting with clinical triad findings were considered Type III. KFS patients with multiple contiguous congenitally fused cervical levels are confirmed to be at a significantly increased risk of severe clinical manifestations. As such, proper precautionary measures and close follow-up should be suggested in KFS patients, especially those regarded as Type III.

Although the pathogenesis of the developmental defect underlying KFS is thought to be attributable to the paraxial mesoderm and somites at the embryonic stage, which may result from mutations or disruptions in genes regulating segmentation and resegmentation, the underlying etiology of KFS is still inconclusive [2, 34, 35]. Previous studies suggested that mutations in *GDF6* [10, 36] and *GDF3* [13] were associated with autosomal dominant KFS. In addition, a truncating mutation in *MEOX1* [11, 12, 37] and a homozygous nonsense mutation in *MYO18B* [14, 38] were identified in autosomal recessive KFS families. Using WES, a homozygous frameshift mutation (c.299delT: p. L100fs) in *RIPPLY2* was confirmed as a novel gene for autosomal recessive KFS in a consanguineous family [15, 39]. In this study, by performing WES on 37 KFS patients, we investigated rare variants in known KFS-related genes, and only three variants in *MYO18B* were detected. However, these novel missense variants in *MYO18B* are still of uncertain significance. Therefore, the etiology of the disease has not been fully explained by the reported mutations associated with KFS.

Gene-based burden analysis has grown into a new approach for discovering genes associated with rare disorders, in which the mutational burden of qualifying variants is compared between case and control subjects for each gene [40, 41]. As a result, we identified five genes (*BAZ1B*, *FREM2*, *VANGL1*, *SUFU* and *KMT2D*) with a significantly increased number of predicted damaging exonic variants in the KFS cohort compared with in-house controls in genetic burden analysis. Pathogenic mutations in *BAZ1B* are frequently associated with Williams-Beuren syndrome, which is a neurodevelopmental disorder characterized by craniofacial dysmorphology, cardiovascular problems, renal abnormalities and musculoskeletal abnormalities [27]. Mutations in *VANGL1* have previously been associated only with caudal regression syndrome, a rare neural tube defect disease with an abnormal development of the vertebral column and spinal cord. Furthermore, a rare case report described a female patient with Williams-Beuren syndrome combined with caudal regression syndrome. In addition to the typical developmental abnormalities, fusion of L4–5 and sacrococcygeal agenesis were reported based on radiologic evaluation [42]. These data reinforced the probability of an important role for *BAZ1B* and *VANGL1* in the pathogenesis of congenital vertebral fusion, broadening the spectrum of known disorders related to these genes.

Missense variants and splice region mutations of *SUFU* were identified in two patients. Aside from the common Joubert syndrome phenotypes such as hyperpnoea, eye movements and developmental retardation, calvarial bone formation has been linked to *SUFU* mutations. Although *SUFU* has not been associated with congenital cervical fusion deformities, it has been reported that *SUFU* is the main negative regulator of the Sonic Hedgehog pathway, and ablation of *SUFU* inhibits the proliferation of osteoprogenitor cells, resulting in failure of calvarial bone formation [43, 44]. Nevertheless, the relationship between *SUFU* and the KFS phenotype still requires further study.

Oligogenic inheritance, characterized by the concurrent effect of two or more distinct genes on the resulting phenotype, was subsequently confirmed to apply to other skeletal deformities such as arthrogryposis [45]. Based on the results from previous studies and our findings, the etiology of congenital cervical fusion is unlikely to be fully explained by a monogenetic model for a fraction of patients. It is possible that mutation burden and combinatorial effects of rare variants in genes that interact genetically in the same biological pathways modify the phenotype of KFS due to synergistic or counteracting effects.

## Conclusions

In conclusion, our study presents clinical features and WES findings from a large cohort of KFS patients to date. Beyond identifying known candidate genes, our analysis highlights five novel rare variants associated with cervical congenital fusion among KFS patients through genetic burden analysis. These results indicate a highly significant enrichment of predicted damaging genes and the potential oligogenic inheritance of KFS.

## Supplementary information

**Additional file 1 Table S1.** List of candidate genes associated with vertebral segmentation defects as well as related diseases **Table S2.** Participants’ demographic and clinical characteristics **Table S3.** Gene burden analysis of rare variants of candidate genes between KFS cases and in-house controls

## Data Availability

All data analyzed during this study is included in this published article. The raw data is available at the corresponding author upon request.

## Abbreviations

KFS: Klippel-Feil syndrome
WES: Whole-exome sequencing
ROM: Range of motion
WBS: Williams-Beuren syndrome
MRI: Magnetic resonance imaging
CT: Computed tomography
OMIM: Online Mendelian Inheritance in Man
SNVs: Single-nucleotide variants
ACMG: American College of Medical Genetics and Genomics
GERP: Genomic Evolutionary Rate Profiling
CADD: Combined Annotation Dependent Depletion
SIFT: Sorting Intolerant Form Tolerant
ESP: Exome Sequencing Project
ExAC: Exome Aggregation Consortium
