# Supplementary material for: The mutational burden and oligogenic inheritance in Klippel-Feil syndrome
Source: BMC Musculoskelet Disord. 2020 Apr 11;21:220. doi: 10.1186/s12891-020-03229-x (PMC7149842; doi:10.1186/s12891-020-03229-x)
Supplement: Supplementary file 1 — Additional file 1 Table S1. List of candidate genes associated with vertebral segmentation defects as well as related diseases Table S2. Participants’ demographic and clinical characteristics Table S3. Gene burden analysis of rare variants of candidate genes between KFS cases and in-house controls [file 12891_2020_3229_MOESM1_ESM.docx]

**Supplementary Material**

This supplemental file was intended for publication as a data supplement. The file includes three tables cited in the manuscript.

The contents include the following:

**Table S1. List of candidate genes associated with vertebral segmentation defects as well as related diseases**

**Table S2. Participants’ demographic and clinical characteristics**

**Table S3. Gene burden analysis of rare variants of candidate genes between KFS cases and in-house controls**

**Table S1** List of candidate genes associated with vertebral segmentation defects as well as related diseases

| **Gene** | **Associated phenotype** |
| --- | --- |
| *ACVR1* | Fibrodysplasia ossificans progressiva, abnormal vertebral morphology |
| *ANKRD11* | KBG syndrome, delayed skeletal maturation, scoliosis, kyphosis, short neck |
| *ATRX* | Alpha-thalassemia/mental retardation syndrome, kyphoscoliosis, short neck |
| *B3GLCT* | Peters-plus syndrome, short neck |
| *BAZ1B* | Williams-Beuren syndrome |
| *BMPER* | Diaphanospondylodysostosis, abnormal vertebral segmentation and fusion |
| *CCDC22* | Ritscher-Schinzel syndrome-2, scoliosis, kyphosis, short neck |
| *CHD7* | CHARGE syndrome, idiopathic scoliosis |
| *CHN1* | Abnormal vertebral segmentation and fusion, short neck, Duane retraction syndrome 2 |
| *CHRM3* | Prune belly syndrome, scoliosis |
| *CHRNA1* | Multiple pterygium syndrome, myasthenic syndrome, vertebral fusion, abnormal cervical curvature |
| *CHRND* | Multiple pterygium syndrome, myasthenic syndrome |
| *CHRNG* | Abnormal cervical curvature, fused cervical vertebrae, vertebral fusion, Escobar syndrome |
| *CLIP2* | Williams-Beuren syndrome |
| *COG1* | Congenital disorder of glycosylation, butterfly vertebrae, short neck, scoliosis |
| *COL25A1* | Abnormal vertebral segmentation and fusion, fibrosis of extraocular muscles, short neck |
| *COL2A1* | Achondrogenesis, spondyloepiphyseal dysplasia, C1-2 subluxation, abnormality of vertebral morphology |
| *CPLX1* | Abnormal morphology of the vertebral bodies, vertebral fusion, epileptic encephalopathy, scoliosis |
| *CTBP1* | HADDTS, vertebral fusion, abnormal form of the vertebral bodies, rib segmentation abnormalities |
| *DLL3* | Spondylocostal dysostosis, vertebral fusion, spina bifida occulta, block vertebrae |
| *EBP* | Chondrodysplasia punctata, MEND syndrome, abnormal vertebral morphology, scoliosis |
| *ELN* | Williams-Beuren Syndrome, Arnold-Chiari malformation, abnormal morphology of the vertebral bodies |
| *FGD1* | Aarskog-Scott syndrome, abnormal vertebral segmentation and fusion, abnormality of the cervical spine |
| *FGFR1* | Hartsfield syndrome, Jackson-Weiss syndrome, short neck |
| *FGFR2* | Apert syndrome, craniofacial-skeletal-dermatological dysplasia, cervical vertebral fusion |
| *FGFRL1* | Vertebral fusion, abnormal morphology of the vertebral bodies |
| *FKRP* | Muscular dystrophy-dystroglycanopathy, spinal deformities |
| *FLNA* | Terminal osseous dysplasia, congenital short bowel syndrome, abnormal bone structure |
| *FLNB* | Atelosteogenesis, Larsen syndrome, spondylocarpotarsal synostosis syndrome, vertebral fusion |
| *FRAS1* | Fraser syndrome 1 |
| *FREM2* | Fraser syndrome 2, cryptophthalmos |
| *FUZ* | Abnormal vertebral segmentation and fusion, neural tube defects, scoliosis |
| *GDF3* | Klippel-Feil syndrome 3 |
| *GDF5* | Acromesomelic dysplasia, brachydactyly, chondrodysplasia, multiple synostoses syndrome 2 |
| *GDF6* | Klippel-Feil syndrome 1, multiple synostoses syndrome 4 |
| *GLI3* | Greig cephalopolysyndactyly syndrome, Pallister-Hall syndrome, polydactyly |
| *GPC3* | Simpson-Golabi-Behmel syndrome, vertebral fusion, short neck |
| *GPC4* | Keipert syndrome, vertebral fusion, scoliosis |
| *GRIP1* | Fraser syndrome 3, syndactyly |
| *GTF2IRD1* | Williams-Beuren syndrome |
| *GTF2I* | Williams-Beuren syndrome, thymic epithelial tumors |
| *HES7* | Spondylocostal dysostosis 4, short neck, rib fusion, finger syndactyly |
| *HGD* | Vertebral fusion, alkaptonuria |
| *HOXD13* | Brachydactyly-syndactyly syndrome, abnormality of the ribs |
| *HRAS* | Costello syndrome, short neck, kyphoscoliosis, Arnold-Chiari malformation |
| *IKBKG* | Kyphoscoliosis, ectodermal dysplasia with immunodeficiency-1 |
| *IL1RN* | Fused cervical vertebrae, osteomyelitis with periostitis and pustulosis |
| *JAG1* | Alagille syndrome 1, butterfly vertebrae |
| *KANSL1* | Koolen-De Vries syndrome, vertebral fusion, scoliosis |
| *KDM6A* | Kabuki syndrome 2, abnormal vertebral morphology, butterfly vertebrae |
| *KMT2D* | Kabuki syndrome 1, scoliosis, abnormal vertebral morphology |
| *KRAS* | Noonan syndrome, short neck |
| *LETM1* | Wolf-Hirschhorn syndrome, abnormal form of the vertebral bodies, rib fusion |
| *LFNG* | Spondylocostal dysostosis 3, short neck, rib fusion, abnormal morphology of the vertebral bodies |
| *LIMK1* | Williams-Beuren syndrome |
| *MAFB* | Multicentric carpotarsal osteolysis syndrome, abnormal vertebral segmentation and fusion |
| *MAP3K7* | Cardiospondylocarpofacial syndrome, fused cervical vertebrae, rib fusion |
| *MBTPS2* | IFAP syndrome, X-linked Olmsted syndrome, abnormal vertebral morphology |
| *MEOX1* | Klippel-Feil syndrome 2 |
| *MESP2* | Spondylocostal dysostosis, vertebral fusion, rib fusion, block vertebrae, short neck |
| *MNX1* | Currarino syndrome |
| *MYH3* | Contractures, pterygia, and variable skeletal fusions syndrome; short neck; vertebral fusion |
| *MYO18B* | Klippel-Feil syndrome 4 |
| *NALCN* | Congenital contractures of the limbs and face, hypotonia, and developmental delay; short neck |
| *NOG* | Multiple synostoses syndrome 1, brachydactyly type B2, fused cervical vertebrae |
| *NOTCH3* | Lateral meningocele syndrome, vertebral fusion |
| *NRAS* | Noonan syndrome 6, kyphoscoliosis |
| *NSD2* | Wolf-Hirschhorn syndrome, rib fusion, vertebral fusion |
| *ORC1* | Meier-Gorlin syndrome 1, abnormality of the ribs |
| *POR* | Antley-Bixler syndrome |
| *PTCH1* | Basal cell nevus syndrome, vertebral fusion, short neck, abnormality of the neck |
| *PTCH2* | Basal cell nevus syndrome, vertebral fusion |
| *PUF60* | Verheij syndrome, vertebral fusion, short neck |
| *RAD21* | Cornelia de Lange syndrome-4, Mungan syndrome |
| *RAP51* | Fused cervical vertebrae, mirror movements 2 |
| *RBM8A* | Thrombocytopenia-absent radius syndrome, fused cervical vertebrae, scoliosis |
| *RFC2* | Williams syndrome |
| *RIPPLY2* | Spondylocostal dysostosis 6, abnormal form of the vertebral bodies, rib fusion, cervical kyphosis |
| *ROR2* | Robinow syndrome, brachydactyly, vertebral fusion, rib fusion, short neck |
| *SALL4* | Duane-radial ray syndrome, vertebral fusion |
| *SEMA3E* | CHARGE syndrome, brachydactyly, scoliosis |
| *SH2B1* | Early-onset obesity-insulin resistance syndrome |
| *SIX6* | Rib fusion, vertebral fusion |
| *SMAD4* | Myhre syndrome |
| *SOX2* | Abnormal vertebral morphology, vertebral fusion, syndromic microphthalmia type 3 |
| *SOX5* | Lamb-Shaffer syndrome, vertebral fusion |
| *SUFU* | Joubert syndrome 32, basal cell nevus syndrome, abnormality of the neck, vertebral fusion |
| *TBL2* | Williams-Beuren syndrome |
| *TBX6* | Spondylocostal dysostosis 5, vertebral fusion, short neck, abnormality of the ribs, syringomyelia |
| *TMCO1* | Craniofacial dysmorphism, skeletal anomalies, and mental retardation syndrome; short neck |
| *TNNI2* | Arthrogryposis, short neck, scoliosis |
| *TNNT3* | Arthrogryposis, short neck, scoliosis |
| *TPM2* | Arthrogryposis, short neck, kyphoscoliosis |
| *VANGL1* | Abnormal vertebral segmentation and fusion, caudal regression syndrome, Arnold-Chiari malformation |
| *WASHC5* | Ritscher-Schinzel syndrome 1, short neck, syndactyly, spastic paraplegia 8 |
| *WNT7A* | Short neck, syndactyly, fuhrmann syndrome |

Abbreviations: HADDTS – hypotonia, ataxia, developmental delay, and tooth enamel defect syndrome

**Table S2** Participants’ demographic and clinical characteristics

| No. | Sex | Comorbidities | Fusion levels | KFSType | Clinical manifestations |
| --- | --- | --- | --- | --- | --- |
| 1 | F | Syringomyelia, PKD | C6-T1 | III | Limited cervical ROM, torticollis |
| 2 | F | Protruding ears | C2-6 | III | Clinical triad |
| 3 | M | None | C4-T1 | III | Limited cervical ROM |
| 4 | F | Syringomyelia, congenital anal atresia, PKD | C7-T1 | I | None |
| 5 | M | None | C2-T1 | III | Limited cervical ROM |
| 6 | M | Syringomyelia | C2-3, C6-T1 | II | None |
| 7 | M | None | C2-4, C6-7 | II | Limited cervical ROM |
| 8 | M | None | C5-6 | I | None |
| 9 | M | Diastematomyelia,  inguinal hernia | C6-7 | I | None |
| 10 | M | None | C2-3 | I | None |
| 11 | F | Basilar invagination, syringomyelia, diastematomyelia,  tethered cord | C1-4 | III | Clinical triad, muscle weakness |
| 12 | M | None | C2-7 | III | Limited cervical ROM, short neck |
| 13 | F | Diastematomyelia,  tethered cord | C3-4 | I | None |
| 14 | M | None | C2-3 | I | None |
| 15 | M | Lower limb discrepancy | C6-T1 | III | None |
| 16 | F | Patent ductus arteriosus | C5-T1 | III | Limited cervical ROM |
| 17 | M | None | C5-7 | III | Torticollis |
| 18 | F | Aplasia of the uterus | C1-6 | III | Clinical triad |
| 19 | F | None | C6-7 | I | Short neck |
| 20 | F | Mitral valve prolapse | C3-4 | I | None |
| 21 | F | Diastematomyelia | C6-T1 | III | None |
| 22 | M | None | C3-4, C6-7 | II | Limited cervical ROM |
| 23 | M | Tetralogy of Fallot,  congenital solitary kidney | C6-7 | I | None |
| 24 | F | None | C6-7 | I | None |
| 25 | F | Patent foramen ovale | C1-T1 | III | Clinical triad, torticollis |
| 26 | F | Syringomyelia, ventricular hypertrophy | C6-7 | I | Short neck, low posterior hairline |
| 27 | M | None | C2-3 | I | Torticollis |
| 28 | F | None | C2-3 | I | Short neck |
| 29 | M | None | C2-7 | III | Limited cervical ROM, Short neck |
| 30 | M | Syringomyelia | C5-7 | III | None |
| 31 | F | Congenital cardiomyopathy | C3-4 | I | Short neck, low posterior hairline |
| 32 | M | Horseshoe kidney | C4-7 | III | Limited cervical ROM |
| 33 | M | None | C3-4 | I | None |
| 34 | F | None | C4-5, C6-7 | II | Limited cervical ROM |
| 35 | F | None | C2-4 | III | Limited cervical ROM |
| 36 | M | None | C4-6 | III | Limited cervical ROM, short neck |
| 37 | M | None | C2-3, C6-T1 | II | Limited cervical ROM |

Abbreviations: PKD – polycystic kidney disease; ROM – range of motion

**Table S3** Gene burden analysis of rare variants of candidate genes between KFS cases and in-house controls

| Gene | KFS allele number | Control allele number | Odds ratio | *P*-value |
| --- | --- | --- | --- | --- |
| *ANKRD11* | 1 | 8 | 1.826 | 0.569 |
| *BAZ1B* | 3 | 1 | 47.029 | 2.32E-08 |
| *CHD7* | 2 | 7 | 4.302 | 0.053 |
| *CHRNG* | 1 | 2 | 7.389 | 0.058 |
| *COG1* | 1 | 3 | 4.917 | 0.131 |
| *FLNA* | 1 | 4 | 3.681 | 0.217 |
| *FRAS1* | 1 | 11 | 1.321 | 0.792 |
| *FREM2* | 3 | 5 | 9.335 | 0.0003 |
| *FUZ* | 1 | 5 | 2.939 | 0.308 |
| *GRIP1* | 1 | 2 | 7.389 | 0.058 |
| *HOXD13* | 1 | 1 | 14.806 | 0.012 |
| *KMT2D* | 3 | 11 | 4.195 | 0.021 |
| *LFNG* | 1 | 1 | 14.806 | 0.012 |
| *MAP3K7* | 1 | 1 | 14.806 | 0.012 |
| *MYH3* | 1 | 4 | 3.681 | 0.217 |
| *MYO18B* | 3 | 14 | 3.277 | 0.058 |
| *POR* | 1 | 2 | 7.389 | 0.058 |
| *ROR2* | 1 | 3 | 4.917 | 0.131 |
| *SUFU* | 2 | 2 | 15.200 | 0.0004 |
| *TBX6* | 1 | 3 | 4.917 | 0.131 |
| *VANGL1* | 2 | 4 | 7.571 | 0.007 |
| *WNT7A* | 1 | 0 | ∞ | 0.0001 |
| *ATRX* | 0 | 1 | 0.000 | 1.000 |
| *BMPER* | 0 | 6 | 0.000 | 1.000 |
| *CCDC22* | 0 | 3 | 0.000 | 1.000 |
| *CHN1* | 0 | 1 | 0.000 | 1.000 |
| *CHRM3* | 0 | 1 | 0.000 | 1.000 |
| *CHRNA1* | 0 | 3 | 0.000 | 1.000 |
| *CHRND* | 0 | 2 | 0.000 | 1.000 |
| *COL2A1* | 0 | 8 | 0.000 | 1.000 |
| *CTBP1* | 0 | 3 | 0.000 | 1.000 |
| *DLL3* | 0 | 1 | 0.000 | 1.000 |
| *ELN* | 0 | 1 | 0.000 | 1.000 |
| *FGFR1* | 0 | 4 | 0.000 | 1.000 |
| *FGFR2* | 0 | 9 | 0.000 | 1.000 |
| *FGFRL1* | 0 | 6 | 0.000 | 1.000 |
| *FLNB* | 0 | 8 | 0.000 | 1.000 |
| *GDF5* | 0 | 2 | 0.000 | 1.000 |
| *HGD* | 0 | 1 | 0.000 | 1.000 |
| *JAG1* | 0 | 4 | 0.000 | 1.000 |
| *KANSL1* | 0 | 1 | 0.000 | 1.000 |
| *LETM1* | 0 | 4 | 0.000 | 1.000 |
| *LIMK1* | 0 | 2 | 0.000 | 1.000 |
| *MBTPS2* | 0 | 1 | 0.000 | 1.000 |
| *MEOX1* | 0 | 2 | 0.000 | 1.000 |
| *NOTCH3* | 0 | 5 | 0.000 | 1.000 |
| *ORC1* | 0 | 2 | 0.000 | 1.000 |
| *PTCH1* | 0 | 3 | 0.000 | 1.000 |
| *PTCH2* | 0 | 4 | 0.000 | 1.000 |
| *RAD21* | 0 | 2 | 0.000 | 1.000 |
| *RIPPLY2* | 0 | 1 | 0.000 | 1.000 |
| *SALL4* | 0 | 8 | 0.000 | 1.000 |
| *SH2B1* | 0 | 2 | 0.000 | 1.000 |
| *SIX6* | 0 | 1 | 0.000 | 1.000 |
| *SMAD4* | 0 | 1 | 0.000 | 1.000 |
| *TBL2* | 0 | 4 | 0.000 | 1.000 |
| *TNNT3* | 0 | 1 | 0.000 | 1.000 |
| Overall | 33 | 202 | 13.559 | 8.29E-10 |

The *P*-values were calculated using Fisher's exact test.
